# **Expanding Public Health Policy Analysis for Transformative Change: The Importance of Power and Ideas** Comment on "What Generates Attention to Health in Trade Policy-Making? Lessons From Success in Tobacco Control and Access to Medicines: A Qualitative Study of Australia and the (Comprehensive and Progressive) Trans-Pacific Partnership"

**DOI:** 10.34172/ijhpm.2020.200

**Published:** 2020-10-18

**Authors:** Penelope Milsom, Richard Smith, Helen Walls

**Affiliations:** ^1^Department of Global Health and Development, Faculty of Public Health and Policy, London School of Hygiene and Tropical Medicine. London, UK.; ^2^College of Medicine and Health, University of Exeter, Exeter, UK.

**Keywords:** Health Policy, Power, Neoliberalism, Trade Policy

## Abstract

It is increasingly recognised within public health scholarship that policy change depends on the nature of the power relations surrounding and embedded within decision-making spaces. It is only through sustained shifts in power in all its forms (visible, hidden and invisible) that previously excluded perspectives have influence in policy decisions. Further, consideration of the underlying neoliberal paradigm is essential for understanding how existing power dynamics and relations have emerged and are sustained. In their analysis of political and governance factors, Townsend et al have provided critical insight into future potential strategies for increasing attention to health concerns in trade policy. In this commentary we explore how incorporating theories of power more rigorously into similar political analyses, as well as more explicit critical consideration of the neoliberal political paradigm, can assist in analysing if and how strategies can effectively challenge existing power relations in ways that are necessary for transformative policy change.

 Understanding the political and governance conditions that enable or constrain attention to non-communicable disease (NCD)-related health issues in trade policy is critical to making progress towards institutionalizing public health impact analysis into trade policy-making. Townsend and colleagues’^1^ comparative analysis of NCD-related issues in Australia during the Trans-Pacific Partnership negotiations makes a significant contribution in this area, offering insight into the process, and a number of potential strategies to increase attention to health in future trade policy in Australia. However, there is considerable scope for this research, like the vast majority of health policy process and governance research, to engage more explicitly with concepts and analysis of power. It is increasingly recognised within public health scholarship that policy change depends on the nature of the power relations surrounding and embedded within decision-making spaces and only through sustained shifts in power can previously excluded perspectives have influence in policy decisions.^2^ We suggest, therefore, that adopting theories of power from the social sciences would enable clearer identification and visibility of different forms of power and their mechanisms to identify strategies for driving transformative change. Secondly, the analysis of Townsend et al elides critical discussion of the underlying dominant political ideology that both underlies and is maintained by the power dynamics and relations in political and public spaces. As such, the analysis and suggested strategies for promoting greater prioritization of health in trade policy risk being confined within the dominant market-oriented, pro-capitalist neoliberal model. With this comes a missed opportunity to imagine and articulate the transformative structural change necessary to re-orientate power relations such that significant and sustainable prioritization of health in trade policy can be realised.

 Like Shiffman and Smith,^3^ Townsend et al conceptualise power as being held by actors, defining it as the ‘strength of the individuals and organisations concerned with the issue, including the cohesiveness of advocacy groups, presence of strong leaders, supportive institutions and strong civil society mobilisation.’ We suggest this definition excludes important structural (hidden) and discursive (invisible) forms of power. It is also somewhat unclear in describing the mechanisms of power (ie, how it actually operates). Finally, it fails to indicate the impact of power in terms of policy action or inaction. Various useful theories and frameworks of power have been developed by social and political science scholars^2,4,5^ and more recently public health academics.^6^ While each has specific strengths, we explore here Gavanta’s Power Cube as one example of a useful analytical tool for understanding policy processes and subsequent policy action or inaction, including, we argue, how and why health issues are not prioritized on trade policy agendas. Further, such frameworks have the potential to help public health policy scholars, practitioners and advocates identify and evaluate if and how different strategies for change in turn change the power relations that currently hold health issues outside the concerns of trade policy-makers.^2^ They can also provide a tool for coordinating complementary actions to challenge power across different spaces (open, closed, invited and claimed) and levels (international, national and sub-national) to more effectively and sustainably transform power relations.^2^

 Building on Lukes’ three dimensions of power,^5^ Gavanta outlines three key forms of power. Visible power refers to observable influence over *decision-making* and includes the visible and definable aspects of political power – formal rules, procedures and authorities of decision-making. For example, most governments do not have formal structures for meaningful involvement of public health actors or civil society in trade policy development while corporate actors generally do, contributing to their significant influence over trade policy decisions. Hidden power refers to controlling the political agenda and involves the processes by which certain powerful people and institutions take advantage of economic structures and institutional practices to limit who is included in decision-making spaces, whose interests are valued and the scope of alternatives for consideration.^4^ For example, as was identified by Townsend et al, alcohol exporting countries tend to neglect alcohol-related public health concerns in the trade domain.

 Invisible power is about shaping interpretation and perceptions of what is acceptable.^2^ It the most insidious and pervasive form of power and tends to be most commonly neglected in health policy and governance research. Invisible power involves shaping the ‘psychological and ideational boundaries’ of participation with significant problems and potential solutions held outside the consciousness of the actors involved, including those directly affected by the problem.^2^ This is achieved through mechanisms of socialisation, including the transfer of culture and ideas, that promote the perception of issues in one way rather than another, ultimately defining what solutions are normal, acceptable and safe.^2^ As such, policy actors can be prevented from elevating solutions in their own real interest^5^. For example, NCDs are widely understood as problems of ‘individual responsibility’ and demand for risk commodities including alcohol and ultra-processed foods (UPF) a matter of choice, not a supply problem facilitated by trade liberalization. As such, nutrition and alcohol-related harm are often not perceived as issues to be considered within trade policy.^7-9^ While actors can have agency over invisible power, it can also operate at the system level. Over time the norm of prioritizing economic interests over food and alcohol-related health issues in trade policy-making have become entrenched and public health strategies that interfere with ‘free’ and open markets are considered radical.

 Neoliberalism can be defined as a policy paradigm focused on reshaping the regulatory environment in favour of free market principles as the most effective means of achieving economic growth and public welfare.^10,11^ Rushton and Williams describe neoliberalism as the ‘deep core’ of the contemporary global political economy providing an overarching logic, set of assumptions and values operating across policy domains. As such they consider neoliberalism to have profoundly affected the configuration of global health policy-making power and authority.^12^ We hold a similar perspective and argue therefore that power relations and dynamics in trade and health policy-making should explicitly be considered within the context of neoliberalism, although specificity, nuance and reflexivity are critical to understanding neoliberalism’s varied manifestations in different political and cultural contexts and for not over-stating its impacts.^13^ Townsend et al do note in their introduction that framings used in trade policy discourse that align with a ‘dominant neoliberal market-oriented discourse’ promotes the privileging of UPF and alcohol companies’ export interests over NCD prevention concerns. They also report in their findings that only framings of access to generic medicines aligned with neoliberal ideas were advanced by trade actors. However, a deeper interrogation of how any of the other various ‘political and governance conditions’ identified in the analysis have themselves been shaped by and generate power relations and dynamics under the neoliberal policy paradigm would be valuable.

 For example, neoliberalism has facilitated the profitability and global expansion of UPF and alcohol corporations and advertising/marketing agencies.^14^ This has given UPF and alcohol corporations the capacity to undertake intensive and sophisticated marketing campaigns embedding their products in different cultures through shifting cultural norms around consumption of their products^15^; and to use various strategies to amplify framing of NCDs that resonate with neoliberal values of freedom and individual choice.^16^ Via these mechanisms corporations capture invisible power influencing the way the public, political parties and trade policy-makers perceive NCDs and the appropriate policy responses as largely limited to individual-level harm reduction interventions. Corporations are also able to take advantage of hidden power under neoliberalism. Given governments’ reliance on private industry profit to achieve the narrow economic growth objective, profitable corporations enjoy close relationships with and access to trade policy-makers and the trade policy agenda is largely shaped by their interests, as was identified by Townsend et al. Most directly, neoliberal logic has shaped the existing international trade and investment agreement context that provides corporations with legal grounds and means to threaten or pursue legal challenges against NCD policy. While this may be a relatively effective mechanism of visible power, as Townsend et al found, trade or investment-related litigation can also generate greater attention to NCD issues in trade policy spaces.

 While the researchers make important recommendations for promoting greater prioritisation of health in trade policy, without incorporating explicit and comprehensive analysis of power and paradigms, their analysis tends to remain for the most part constrained within the existing structures of neoliberalism and the associated power structure. For example, they propose mandatory health impact assessments of proposed trade texts and further studies to collect the evidence on the causal connections between NCD risk products and trade deals. While these are important considerations, we also know that instrumental power alone, for example using public health knowledge and evidence to encourage policy action, is alone usually insufficient to drive public policy change. For evidence to drive policy action is must also generally be confluent with current political discourse and ideological conditions.^17^

 The authors also call for greater trade literacy amongst the health community, including health policy-makers, as well as institutional changes that ensure participation of these health actors in trade negotiation processes. But participation does not necessarily translate into sharing power, including hidden (agenda-setting) power. For example, after pressure during the Trans-Pacific Partnership negotiations, the US government proposed establishing a Public Interest Trade Advisory Committee to input into negotiations. However, Public Interest Trade Advisory Committee members would be bound by nondisclosure agreements, restricting their engagement with the wider civil society movement and be located in less influential groups than industry representatives.^18^ In Thailand, despite improving health policy-maker’s trade literacy and increasing public health actor participation in trade policy spaces, it is not clear that these changes actually influenced Thailand’s trade negotiating positions.^19^ We suggest that these examples indicate that without transforming the overarching policy paradigm, the existing dynamics of hidden power in trade policy spaces are simply reproduced, albeit more subtly, within new institutional arrangements.

 Finally, we strongly agree that the potential wide-ranging impacts of trade agreements on the social determinants of health, including environment, employment, human rights and social sectors, provides critical opportunities for a broader coalition of public interest actors. But what strategies can these public interest actors use to effectively challenge existing power relations and how much can they achieve under the existing paradigmatic constraining goals and values? We suggest such a coalition may be most effective in advancing shared interests in trade policy if it coalesced around an alternative paradigm with new values and goals. A paradigm that, for example, is designed to reshape rather than grow the economy and aims to meet the health and social needs of the population within the means of the planet.^20,21^ A broad coalition of public interest actors could then, for example, begin to claim back some invisible power by disseminating framings that resonate with their new shared ideology, expanding the ideational boundaries around how issues are interpreted and future possibilities for addressing them. For example, decoupling the neoliberal association of free markets with free choice, and communicating a more nuanced understanding of the complex structural and social forces interacting to regulate behaviour can expand the boundaries around conceivable policy options to include those that would genuinely support informed choices and create environments in which health products are affordable and accessible.^20^

 We acknowledge though that driving paradigmatic change is an enormously challenging undertaking, especially for less powerful actors. Theories proposed by some scholars indicate that relatively smaller-scale strategies, like many suggested Townsend et al, can generate cumulative healthy adjustments to and additional health protective measures within trade policy which eventually can lead to the necessary broader paradigmatic transformation.^22^ However, we would again caution that if such strategies do not effectively challenge all existing forms of power that maintain current trade policy-making norms where trade is often prioritized over health, they are unlikely to lead to transformative change.

 Through their analysis of political and governance factors, Townsend et al have provided critical insight into future potential strategies for increasing attention to health concerns in trade policy. We propose future research builds on this and other similar political analyses to adopt a more in-depth conceptualization and analysis of power with explicit critical reflection of the paradigmatic context shaping the power relations between trade, public health and corporate actors. Using such an approach may facilitate the discovery and development of potential strategies to tackle the deep structural causes of NCD risk exposure.

## Ethical issues

 Not applicable.

## Competing interests

 Authors declare that they have no competing interests.

## Authors’ contributions

 PM designed the commentary with input from RS and HW. PM drafted the manuscript. Critical revisions of the manuscript for important intellectual content were conducted by all authors.

## Funding

 This work was supported by doctoral funding from the Wellcome Trust [grant number 203286/Z/16/Z] received by PM.

## Authors’ affiliations

^1^Department of Global Health and Development, Faculty of Public Health and Policy, London School of Hygiene and Tropical Medicine. London, UK. ^2^College of Medicine and Health, University of Exeter, Exeter, UK.
